# Supplementary material for: Differential Responses of OsMPKs in IR56 Rice to Two BPH Populations of Different Virulence Levels
Source: Int J Mol Sci. 2018 Dec 13;19(12):4030. doi: 10.3390/ijms19124030 (PMC6320944; doi:10.3390/ijms19124030)
Supplement: Supplementary file 1 [file ijms-19-04030-s001.zip › ijms-384944 to proofreading supplementary/Supplementary Tables.docx]

**Table S1. The three-way ANOVA analysis results of the expression patterns of 17 *OsMPK*s in both compatible and incompatible interaction.**

| **Genes** | **Source of Variance** | **Main effects** | | |  | **Interaction effects** | | | | **Error** |
| --- | --- | --- | --- | --- | --- | --- | --- | --- | --- | --- |
|  |  | Rice varieties | BPH populations | Days post infestation |  | Rice varieties × BPH populations | Rice varieties × Days post infestation | BPH populations × Days post infestation | Rice varieties × BPH populations ×Days post infestation |  |
|  | df | 1 | 2 | 2 |  | 2 | 2 | 4 | 4 | 36 |
| *OsMPK6* | F value | 0.12 | 0.02 | 0.22 |  | 0.39 | 0.27 | 0.11 | 0.31 |  |
|  | P value | 0.73 | 0.98 | 0.81 |  | 0.68 | 0.76 | 0.98 | 0.87 |  |
| *OsMPK10* | F value | 0.15 | 0.02 | 0.37 |  | 0.58 | 0.46 | 0.29 | 0.3 |  |
|  | P value | 0.7 | 0.99 | 0.69 |  | 0.56 | 0.64 | 0.88 | 0.88 |  |
| *OsMPK11* | F value | 0.41 | 2.24 | 0.31 |  | 0.64 | 1.2 | 0.73 | 1.62 |  |
|  | P value | 0.53 | 0.12 | 0.74 |  | 0.53 | 0.31 | 0.58 | 0.19 |  |
| *OsMPK15* | F value | 0.46 | 0.11 | 1.43 |  | 0.06 | 0.22 | 0.37 | 0.08 |  |
|  | P value | 0.5 | 0.89 | 0.25 |  | 0.94 | 0.81 | 0.83 | 0.99 |  |
| *OsMPK17* | F value | 1.43 | 2.03 | 1.45 |  | 0.42 | 0.4 | 0.89 | 0.34 |  |
|  | P value | 0.24 | 0.15 | 0.25 |  | 0.66 | 0.67 | 0.48 | 0.85 |  |
| *OsMPK3* | F value | 12.04** | 6.44** | 13.93** |  | 9.41** | 9.44** | 11.12** | 11.41** |  |
|  | P value | **<0.0001** | **<0.0001** | **<0.0001** |  | **<0.0001** | **<0.0001** | **<0.0001** | **<0.0001** |  |
| *OsMPK5* | F value | 10.53** | 112.52** | 164.48** |  | 99.25** | 24.96** | 41.21** | 34.36** |  |
|  | P value | **<0.0001** | **<0.0001** | **<0.0001** |  | **<0.0001** | **<0.0001** | **<0.0001** | **<0.0001** |  |
| *OsMPK7* | F value | 99.32** | 73.58** | 31.99** |  | 96.14** | 31.99** | 27.74** | 33.08** |  |
|  | P value | **<0.0001** | **<0.0001** | **<0.0001** |  | **<0.0001** | **<0.0001** | **<0.0001** | **<0.0001** |  |
| *OsMPK12* | F value | 46.85** | 62.7** | 38.84** |  | 42.7** | 23.45** | 23.35** | 20.9** |  |
|  | P value | **<0.0001** | **<0.0001** | **<0.0001** |  | **<0.0001** | **<0.0001** | **<0.0001** | **<0.0001** |  |
| *OsMPK13* | F value | 21.74** | 81.49** | 21.89** |  | 56.18** | 6.66** | 21.72** | 15.23** |  |
|  | P value | **<0.0001** | **<0.0001** | **<0.0001** |  | **<0.0001** | **<0.0001** | **<0.0001** | **<0.0001** |  |
| *OsMPK14* | F value | 48.8** | 32.22** | 12.73** |  | 41.62** | 16.05** | 8.94** | 11.71** |  |
|  | P value | **<0.0001** | **<0.0001** | **<0.0001** |  | **<0.0001** | **<0.0001** | **<0.0001** | **<0.0001** |  |
| *OsMPK16* | F value | 36.12** | 27.46** | 24.83** |  | 33.2** | 26.55** | 18.1** | 21.85** |  |
|  | P value | **<0.0001** | **<0.0001** | **<0.0001** |  | **<0.0001** | **<0.0001** | **<0.0001** | **<0.0001** |  |
| *OsMPK1* | F value | 2.64 | 4.48* | 8.43** |  | 2.25 | 2.46 | 5.06** | 3.78** |  |
|  | P value | 0.11 | **0.02** | **<0.0001** |  | 0.12 | 0.1 | **<0.0001** | **0.01** |  |
| *OsMPK4* | F value | 0.21 | 13.98** | 16.61** |  | 46.07** | 0.11 | 5.25 | 14.83 |  |
|  | P value | 0.65 | **<0.0001** | **<0.0001** |  | **<0.0001** | 0.89 | <0.0001 | <0.0001 |  |
| *OsMPK9* | F value | 2.57 | 9.32** | 16.32** |  | 20.6** | 0.67** | 5.03** | 12.57** |  |
|  | P value | 0.12 | **<0.0001** | **<0.0001** |  | **<0.0001** | 0.52 | **<0.0001** | **<0.0001** |  |
| *OsMPK8* | F value | 1.69 | 3.08 | 15.67** |  | 8.78** | 1.91 | 4.48** | 9.08** |  |
|  | P value | 0.2 | 0.06 | **<0.0001** |  | **<0.0001** | 0.16 | **<0.0001** | **<0.0001** |  |
| *OsMPK2* | F value | 1.01 | 2.47 | 2.73 |  | 1.08 | 6.49** | 1.5 | 2.8* |  |
|  | P value | 0.32 | 0.1 | 0.08 |  | 0.35 | **<0.0001** | 0.22 | **0.04** |  |

**Table S2. Two-way ANOVA analysis of the expression patterns of signaling genes, phenylpropanoid biosynthesis genes, ROS-responsive genes, *OsLecRK*s, and activity of ROS-responsive enzymes, respectively.**

| **Genes** | **Source of Variance** | **Day 1** | | | **Error** |  | **Day 3** | | | **Error** |
| --- | --- | --- | --- | --- | --- | --- | --- | --- | --- | --- |
|  |  | **Main effects** | | **Interaction effect** |  |  | **Main effects** | | **Interaction effect** |  |
|  |  | **Rice varieties** | **BPH populations** | **Rice varieties × BPH populations** |  |  | **Rice varieties** | **BPH populations** | **Rice varieties × BPH populations** |  |
|  | **df** | 1 | 2 | 2 | 12 |  | 2 | 2 | 4 | 12 |
| **SA signaling genes** | | | | | | | | | | |
| *OsPAL1* | F value | 11.746** | 9.627** | 11.908** |  |  | 124.59** | 97.147** | 99.034** |  |
|  | P value | **0.005** | **0.0032** | **0.0014** |  |  | **<0.0001** | **<0.0001** | **<0.0001** |  |
| *OsEDS1* | F value | 14.03** | 9.051** | 6.995** |  |  | 57.41** | 100.659** | 62.125** |  |
|  | P value | **0.0028** | **0.004** | **0.0097** |  |  | **<0.0001** | **<0.0001** | **<0.0001** |  |
| *OsNPR1* | F value | 3.495 | 4.284* | 2.613 |  |  | 0.746 | 6.542* | 0.609 |  |
|  | P value | 0.0861 | **0.0395** | 0.1142 |  |  | 0.4048 | **0.012** | 0.56 |  |
| **JA signaling genes** | | | | | | | | | | |
| *OsAOS2* | F value | 26.364** | 33.247** | 39.426** |  |  | 14.381** | 5.238* | 8.02** |  |
|  | P value | **0.0002** | **<0.0001** | **<0.0001** |  |  | **0.0026** | **0.0232** | **0.0061** |  |
| *OsLOX1* | F value | 16.653** | 16.229** | 23.055** |  |  | 7.609* | 1.275 | 2.108 |  |
|  | P value | **0.0015** | **0.0004** | **0.0001** |  |  | **0.0173** | 0.3146 | 0.1643 |  |
| *OsAOS1* | F value | 4.611 | 4.199* | 7.452 |  |  | 7.313* | 0.8 | 2.713 |  |
|  | P value | 0.0529 | **0.0415** | 0.0079 |  |  | **0.0192** | 0.4718 | 0.1066 |  |
|  | | | | | | | | | | |
| **ET signaling genes** | | | | | | | | | | |
| *OsASC2* | F value | 9.769** | 12.937** | 7.462** |  |  | 45.868** | 32.434** | 41.621** |  |
|  | P value | **0.0088** | **0.001** | **0.0078** |  |  | **<0.0001** | **<0.0001** | **<0.0001** |  |
| *OsEIN2* | F value | 15.644** | 11.004** | 14.369** |  |  | 108.273** | 143.008** | 53.717** |  |
|  | P value | **0.0019** | **0.0019** | **0.0007** |  |  | **<0.0001** | **<0.0001** | **<0.0001** |  |
| *OsERF3* | F value | 180.537** | 118.484** | 119.707** |  |  | 539.876** | 316.449** | 276.603** |  |
|  | P value | **<0.0001** | **<0.0001** | **<0.0001** |  |  | **<0.0001** | **<0.0001** | **<0.0001** |  |
| **P.P. pathway genes** | | | | | | | | | | |
| *OsC4H* | F value | 46.235** | 51.435** | 59.927** |  |  | 38.158** | 31.992** | 26.147** |  |
|  | P value | **<0.0001** | **<0.0001** | **<0.0001** |  |  | **<0.0001** | **<0.0001** | **<0.0001** |  |
| *OsCHS* | F value | 174.248** | 139.073** | 162.644** |  |  | 123.326** | 65.042** | 109.953** |  |
|  | P value | **<0.0001** | **<0.0001** | **<0.0001** |  |  | **<0.0001** | **<0.0001** | **<0.0001** |  |
| *OsCHI* | F value | 45.47** | 49.316** | 54.086** |  |  | 66.68** | 14.641** | 37.38** |  |
|  | P value | **<0.0001** | **<0.0001** | **<0.0001** |  |  | **<0.0001** | **0.0006** | **<0.0001** |  |
| **ROS-responsive genes** | | | | | | | | | | |
| *OsSOD* | F value | 1.135 | 25.132** | 0.795 |  |  | 0.361** | 3.865** | 0.305** |  |
|  | P value | 0.3076 | **0.0001** | 0.4741 |  |  | **<0.0001** | **<0.0001** | **<0.0001** |  |
| *OsGST* | F value | 0.469 | 18.953** | 1.378 |  |  | 0.147 | 7.262** | 0.098 |  |
|  | P value | 0.5064 | **0.0002** | 0.2892 |  |  | 0.7077 | **0.0086** | 0.9075 |  |
| ***OsLecRK* genes** | | | | | | | | | | |
| *OsLecRK3* | F value | 54.215** | 52.515** | 51.958** |  |  | 95.635** | 74.746** | 56.221** |  |
|  | P value | **<0.0001** | **<0.0001** | **<0.0001** |  |  | **<0.0001** | **<0.0001** | **<0.0001** |  |
| *OsLecRK4* | F value | 136.851** | 109.936** | 115.12** |  |  | 150.813** | 135.763** | 115.739** |  |
|  | P value | **<0.0001** | **<0.0001** | **<0.0001** |  |  | **<0.0001** | **<0.0001** | **<0.0001** |  |
| *OsLecRK1* | F value | 0.018 | 0.446 | 0.013 |  |  | 1.922 | 1.273 | 1.378 |  |
|  | P value | 0.8945 | 0.6506 | 0.9875 |  |  | 0.1909 | 0.3153 | 0.2893 |  |
| **ROS-related enzymes** | | | | | | | | | | |
| POD | F value | 147.183** | 102.978** | 24.938** |  |  | 2.287 | 10.651** | 3.274 |  |
|  | P value | **<0.0001** | **<0.0001** | **0.0001** |  |  | 0.1563 | **0.0022** | 0.0733 |  |
| GST | F value | 320.88** | 133.749** | 87.804** |  |  | 2.089 | 83.922** | 1.689 |  |
|  | P value | **<0.0001** | **<0.0001** | **<0.0001** |  |  | 0.1739 | **<0.0001** | 0.2257 |  |
| SOD | F value | 0.169 | 28.132** | 5.938* |  |  | 123.326** | 65.042** | 109.953** |  |
|  | P value | 0.6886 | **<0.0001** | **0.0161** |  |  | **<0.0001** | **<0.0001** | **<0.0001** |  |
| CAT | F value | 0.195 | 8.422** | 0.012 |  |  | 0.025 | 8.035** | 0.287 |  |
|  | P value | 0.6669 | **0.0052** | 0.9882 |  |  | 0.8778 | **0.0061** | 0.7555 |  |

**Table S3. Two-way ANOVA analysis of the expression patterns of BPH-induced *OsMPK*s post-external SA treatment.**

| **Genes** | **Source of Variance** | **Main effects** | | **Interaction effect** | **Error** |
| --- | --- | --- | --- | --- | --- |
|  |  | **Rice varieties** | **Time post treatment** | **Rice varieties × Time post treatment** |  |
|  | **df** | 1 | 2 | 2 | 12 |
| *OsMPK4* | F value | 14.356** | 35.202** | 7.325** |  |
|  | P value | **0.0026** | **<0.0001** | **0.0083** |  |
| *OsMPK9* | F value | 13.147** | 3.932* | 5.338* |  |
|  | P value | **0.0035** | **0.0486** | **0.022** |  |
| *OsMPK12* | F value | 22.622** | 7.827** | 5.507* |  |
|  | P value | **0.0005** | **0.0067** | **0.0201** |  |
| *OsMPK16* | F value | 24.738** | 17.45** | 22.742** |  |
|  | P value | **0.0003** | **0.0003** | **0.0001** |  |
| *OsMPK14* | F value | 5.422* | 4.54* | 2.286 |  |
|  | P value | **0.0382** | **0.034** | 0.1442 |  |
| *OsMPK5* | F value | 4.201 | 40.983** | 2.704 |  |
|  | P value | 0.0629 | **<0.0001** | 0.1073 |  |
| *OsMPK1* | F value | 0.297 | 0.075 | 0.134 |  |
|  | P value | 0.596 | 0.9285 | 0.8756 |  |
| *OsMPK3* | F value | 0.049 | 0.71 | 0.176 |  |
|  | P value | 0.829 | 0.5114 | 0.8404 |  |
| *OsMPK13* | F value | 0.344 | 0.15 | 0.531 |  |
|  | P value | 0.5687 | 0.8624 | 0.601 |  |
| *OsMPK7* | F value | 4.222 | 1.056 | 1.313 |  |
|  | P value | 0.0623 | 0.378 | 0.305 |  |
| *OsMPK8* | F value | 0.06 | 0.589 | 0.103 |  |
|  | P value | 0.8109 | 0.5702 | 0.9031 |  |

**Table S4. Two-way ANOVA analysis of the expression patterns of BPH-induced *OsMPK*s post-external MeJA treatment.**

| **Genes** | **Source of Variance** | **Main effects** | | **Interaction effect** | **Error** |
| --- | --- | --- | --- | --- | --- |
|  |  | **Rice varieties** | **Time post treatment** | **Rice varieties × Time post treatment** |  |
|  | df | 1 | 2 | 2 | 12 |
| *OsMPK1* | F value | 9.884** | 7.161** | 9.767** |  |
|  | P value | **0.0085** | **0.009** | **0.003** |  |
| *OsMPK4* | F value | 23.248** | 9.538** | 11.681** |  |
|  | P value | **0.0004** | **0.0033** | **0.0015** |  |
| *OsMPK3* | F value | 1.475 | 60.685** | 1.941 |  |
|  | P value | 0.2479 | **<0.0001** | 0.186 |  |
| *OsMPK5* | F value | 1.762 | 19.81** | 0.772 |  |
|  | P value | 0.2091 | **0.0002** | 0.4838 |  |
| *OsMPK7* | F value | 3.354 | 5.815* | 0.722 |  |
|  | P value | 0.092 | **0.0171** | 0.5056 |  |
| *OsMPK13* | F value | 4.624 | 13.823** | 1.891 |  |
|  | P value | 0.0526 | **0.0008** | 0.1932 |  |
| *OsMPK8* | F value | 0.406 | 1.175 | 0.346 |  |
|  | P value | 0.5358 | 0.3419 | 0.7145 |  |
| *OsMPK9* | F value | 3.454 | 3.836 | 0.83 |  |
|  | P value | 0.0878 | 0.0515 | 0.4596 |  |
| *OsMPK12* | F value | 1.602 | 0.291 | 0.263 |  |
|  | P value | 0.2296 | 0.7525 | 0.7734 |  |
| *OsMPK14* | F value | 1.855 | 1.478 | 0.503 |  |
|  | P value | 0.1982 | 0.2669 | 0.6171 |  |
| *OsMPK16* | F value | 0.127 | 0.478 | 0.009 |  |
|  | P value | 0.7283 | 0.6311 | 0.9912 |  |

**Table S5. Two-way ANOVA analysis of the expression patterns of BPH-induced *OsMPK*s post-external ethephon (ET) treatment.**

| **Genes** | **Source of Variance** | **Main effects** | | **Interaction effect** | **Error** |
| --- | --- | --- | --- | --- | --- |
|  |  | **Rice varieties** | **Time post treatment** | **Rice varieties × Time post treatment** |  |
|  | df | 1 | 2 | 2 | 12 |
| *OsMPK4* | F value | 75.835** | 48.515** | 42.123** |  |
|  | P value | **<0.0001** | **<0.0001** | **<0.0001** |  |
| *OsMPK7* | F value | 35.356** | 22.239** | 24.967** |  |
|  | P value | **0.0001** | **0.0001** | **0.0001** |  |
| *OsMPK9* | F value | 33.3** | 58.377** | 19.471** |  |
|  | P value | **0.0001** | **<0.0001** | **0.0002** |  |
| *OsMPK13* | F value | 35.655** | 20.099** | 21.203** |  |
|  | P value | **0.0001** | **0.0001** | **0.0001** |  |
| *OsMPK16* | F value | 14.636** | 15.919** | 14.42** |  |
|  | P value | **0.0024** | **0.0004** | **0.0006** |  |
| *OsMPK14* | F value | 4.721 | 14.016** | 4.691* |  |
|  | P value | 0.0505 | **0.0007** | **0.0312** |  |
| *OsMPK12* | F value | 4.134 | 79.956** | 1.149 |  |
|  | P value | 0.0647 | **<0.0001** | 0.3495 |  |
| *OsMPK1* | F value | 0.553 | 0.866 | 0.282 |  |
|  | P value | 0.4712 | 0.4452 | 0.7592 |  |
| *OsMPK3* | F value | 1.188 | 0.984 | 0.983 |  |
|  | P value | 0.2972 | 0.4021 | 0.4024 |  |
| *OsMPK5* | F value | 1.42 | 0.183 | 0.6 |  |
|  | P value | 0.2564 | 0.8347 | 0.5643 |  |
| *OsMPK8* | F value | 1.822 | 1.007 | 1.561 |  |
|  | P value | 0.202 | 0.3941 | 0.2497 |  |

**Table S6. List of the primers used in qPCR.**

| **Gene name** | **RAP-DB/ Accession number** | **Forward primer (5’-3’)** | **Reverse primer (5’-3’)** |
| --- | --- | --- | --- |
| ***OsMPKs*** |  |  |  |
| *OsMPK1* | Os06g06090 | CATCGCGATCTGAAACCGAG | CGGTTTGCGATCCATCAGTT |
| *OsMPK2* | Os08g06060 | TCTTGCACAGAGACCTCAGG | GCAGCCTACCGACCAGATAT |
| *OsMPK3* | Os02g05480 | TCTCGGGGAAATGGCGATCA | CGATGGGCTTGATTGGCACA |
| *OsMPK4* | Os06g48590 | GCGACAATTACGGAACCTCC | GTGCTTGCGGGTACATACTG |
| *OsMPK5* | Os03g17700 | GAGGGAGATGGTGGCGATAA | CTTGGTTGGAGCGGATGATG |
| *OsMPK6* | Os10g38950 | AGTACCCAAGGCAGGACTTC | GGCAGGTGGGTTCTTCATTG |
| *OsMPK7* | Os05g49140 | GGCGAGGCTTGGATTTTCTC | TTTGTATCGGCTGGAGTCACC |
| *OsMPK8* | Os01g47530 | TGGCGGTGTGTGTTTTCTTT | TTAGTGGTGCGGTCAGTTCT |
| *OsMPK9* | Os05g50560 | CCCGGGTTCGGAATGAGAAA | CTCTTCAGCAGTTGGACGGT |
| *OsMPK10* | Os01g43910 | TTGAGTTGGGAGTCCTTCGC | GGTGCGGTCAACTTTTGCAT |
| *OsMPK11* | Os06g26340 | TGAGGAGGAGAAGAAGGGGA | ATCCACCTCTGCTGCATTCT |
| *OsMPK12* | Os06g49430 | CGACAGTTTGCACACCTTGA | CTGCACTCCTCTCTTGGTCA |
| *OsMPK13* | Os02g04230 | CGGCCATGATCTTGATGCAA | ACAAGCCGAGTTGCCAAATT |
| *OsMPK14* | Os05g05160 | AGTACGGTGAGGGAAACAGG | GCGAAGTATCCGTGTTGCAT |
| *OsMPK15* | Os11g17080 | AACGCATGCTGAGCTTTGAA | TCTTCTTTGGTAATGCGGCG |
| *OsMPK16* | Os01g45620 | GAGTTGTGCGGCTCTTTCTT | GGAGAGCCAAGTAAGTCGGT |
| *OsMPK17* | Os05g50120 | GCTTGAACGCCTTCTTGCTT | GTTCGCGTTCAGAGTTTGCT |
| ***OsLecRKs*** |  |  |  |
| *OsLecRK1* | KF748957 | TTGGCGTGTCTGGAAGGTAT | TCCAGACCGCATTAGTTCCT |
| *OsLecRK3* | KF748973 | CTTGGAGAACGGCATTTGT | TGTCAGGAGAACCCAGCAT |
| *OsLecRK4* | KF748981 | ATCTGGGTCAAGCGTAG | CCAATGGCAGGTTTCT |
| *OsAOS1* | Os03g0767000 | CGAGCTCTTCCTCCGATACG | GTCAGAAGGTGGCCTTCTTGAG |
| *OsAOS2* | Os03t0225900 | CTCGTCGGAAGGCTGTTGCT | ACGATTGACGGCGGAGGTT |
| *OsLOX1* | Os02g0194700 | GGAGGTTCAACGAGAGGATG | GATCCTTGTTCCGGCAGTC |
| *OsPAL1* | Os02g41630 | GCACATCTTGGAGGGAAGCT | GCGCGGATAACCTCAATTTG |
| *OsEDS1* | Os09t0392100 | CATTCCAAGAACGAGGACACT | CAAGACTCAAGGCTAGAACCGA |
| *OsNPR1* | Os01t0194300 | TTTCCGATGGAGGCAAGAG | GCTGTCATCCGAGCTAAGTGTT |
| *OsACS2* | Os04g0578000 | CACCCCGAGGCATCCAT | ATTGGCGATCCTCTTGAACTG |
| *OsEIN2* | Os07g06130 | CAAGGAACCAGTGACAACCA | GCAGTCGTCTCCGCAGTTAG |
| *OsERF3* | Os02g0202000 | GTTCGCTTTCCTTTCAGAGGAT | GCAGCCTGCTCATAGAAAAAGT |
| *OsC4H* | Os05g0320700 | CTCGTCCAGAGCTTCGACCT | GGATCTGGTTGCTGAACTGG |
| *OsCHS* | Os11g0530600 | AGGGAAGAATGGGGACTGAT | TGCCTCGAACTAGCATTCCT |
| *OsCHI* | Os03g0819600 | AGCTCCTGAAGGCGGAAT | GATTTTCACGCGGACACC |
| *OsGST* | Os01t0369700-01 | GTAGGCTCGCCGAGTACG | CAGCTGCTGCCCACTCTG |
| *OsSOD* | Os04g0573200 | CGATCCTGATGATCTTGGAAA | CAGCCTTGAAGTCCGATGAT |
| *OsUbq* | XM_015757177 | GTTCGCCCAGTTGACATCTC | CAGATTGTTGAGGTTAGTATTGC |
