# Supplementary figures and images for: Differential Responses of OsMPKs in IR56 Rice to Two BPH Populations of Different Virulence Levels
Source: Int J Mol Sci. 2018 Dec 13;19(12):4030. doi: 10.3390/ijms19124030 (PMC6320944; doi:10.3390/ijms19124030)

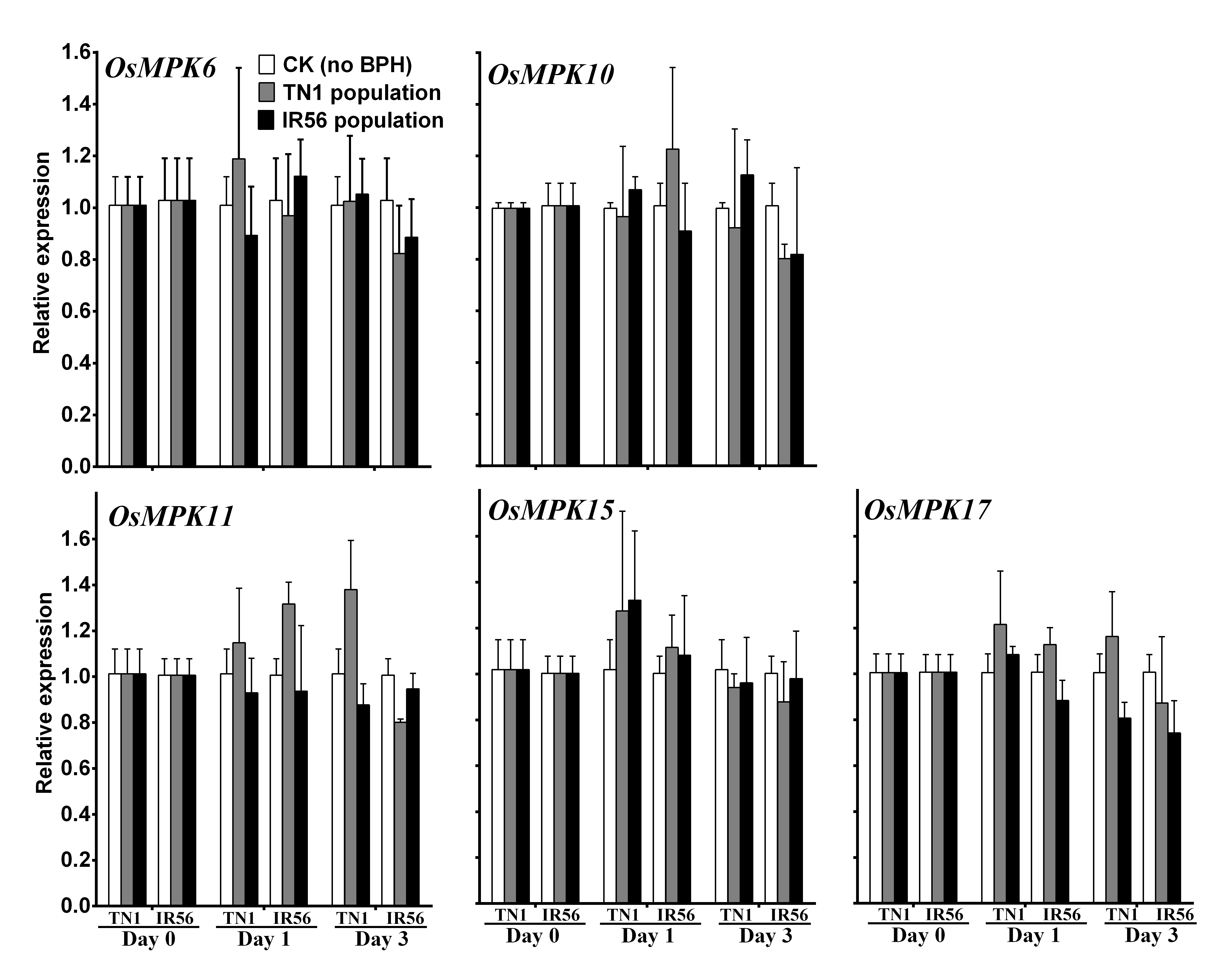

Supplement: Supplementary file 1 [file ijms-19-04030-s001.zip › ijms-384944 to proofreading supplementary/Figure S1.jpg]

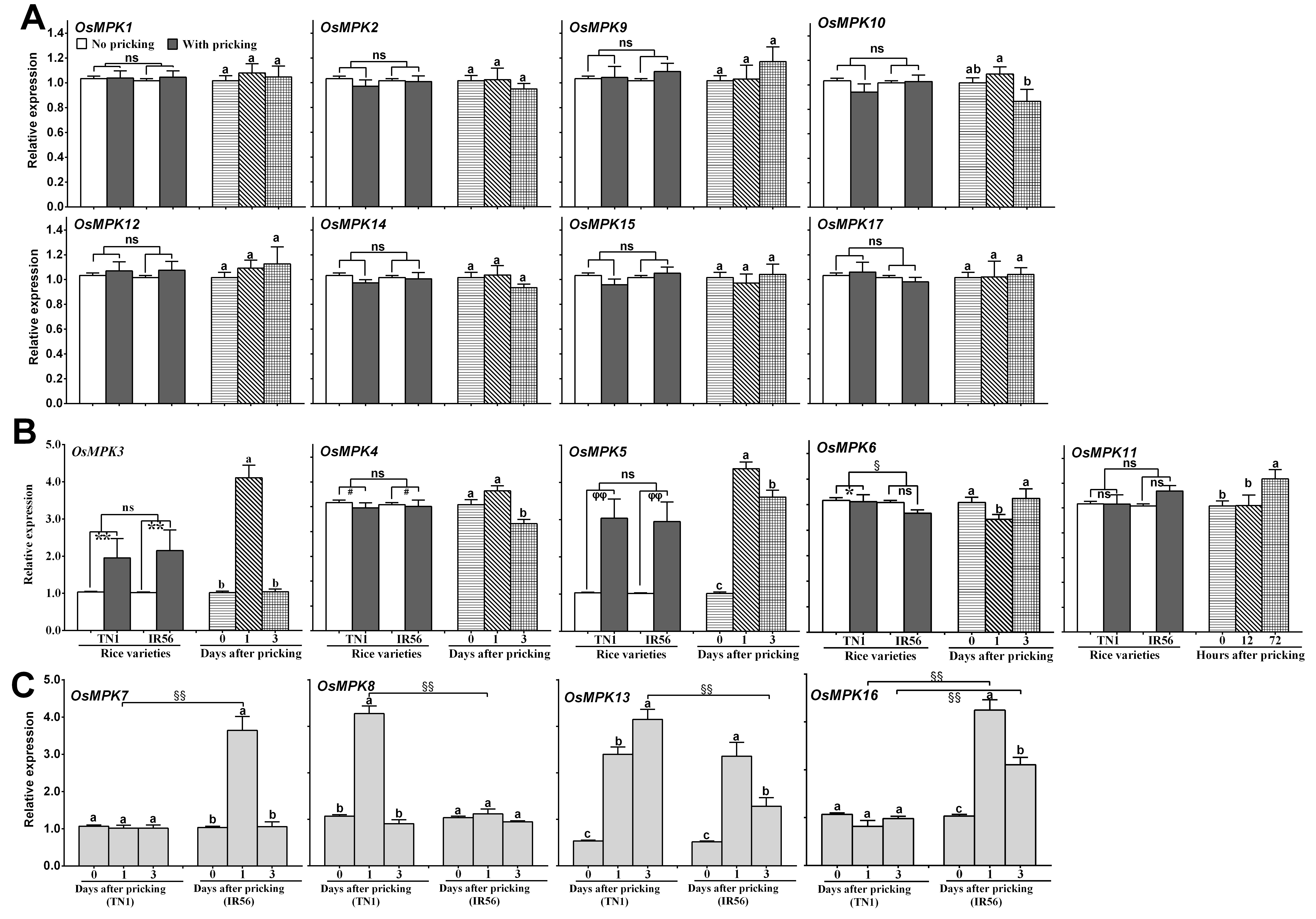

Supplement: Supplementary file 1 [file ijms-19-04030-s001.zip › ijms-384944 to proofreading supplementary/Figure S2.jpg]
